# Whole-genome identification and expression profiling of growth-regulating factor (*GRF*) and *GRF*-interacting factor (*GIF*) gene families in *Panax ginseng*

**DOI:** 10.1186/s12864-023-09435-w

**Published:** 2023-06-16

**Authors:** Ping Wang, Ying Xiao, Min Yan, Yan Yan, Xiujuan Lei, Peng Di, Yingping Wang

**Affiliations:** 1 State Local Joint Engineering Research Centre of Ginseng Breeding and Application, Jilin Agricultural University, Changchun, 130118 China; 2 The SATCM Key Laboratory for New Resources & Quality Evaluation of Chinese Medicine, Institute of Chinese Materia Medica, Shanghai University of Traditional Chinese Medicine, Shanghai, 201203 China

**Keywords:** Growth-regulating factor, *GRF*-interacting factor, *Panax ginseng*, Expression pattern, Cis-acting elements

## Abstract

**Background:**

*Panax ginseng* is a perennial herb and one of the most widely used traditional medicines in China. During its long growth period, it is affected by various environmental factors. Past studies have shown that growth-regulating factors (*GRFs*) and *GRF*-interacting factors (*GIFs*) are involved in regulating plant growth and development, responding to environmental stress, and responding to the induction of exogenous hormones. However, *GRF* and *GIF* transcription factors in ginseng have not been reported.

**Results:**

In this study, 20 *GRF* gene members of ginseng were systematically identified and found to be distributed on 13 chromosomes. The ginseng *GIF* gene family has only ten members, which are distributed on ten chromosomes. Phylogenetic analysis divided these *PgGRFs* into six clades and *PgGIFs* into two clades. In total, 18 of the 20 *PgGRFs* and eight of the ten *PgGIFs* are segmental duplications. Most *PgGRF* and *PgGIF* gene promoters contain some hormone- and stress- related cis-regulatory elements. Based on the available public RNA-Seq data, the expression patterns of *PgGRF* and *PgGIF* genes were analysed from 14 different tissues. The responses of the *PgGRF* gene to different hormones (6-BA, ABA, GA3, IAA) and abiotic stresses (cold, heat, drought, and salt) were studied. The expression of the *PgGRF* gene was significantly upregulated under GA3 induction and three weeks of heat treatment. The expression level of the *PgGIF* gene changed only slightly after one week of heat treatment.

**Conclusions:**

The results of this study may be helpful for further study of the function of *PgGRF* and *PgGIF* genes and lay a foundation for further study of their role in the growth and development of *Panax ginseng*.

**Supplementary Information:**

The online version contains supplementary material available at 10.1186/s12864-023-09435-w.

## Background

Growth-regulating factors (*GRFs*) are unique plant transcription factors that play vital roles in regulating plant growth and development, as well as abiotic stress response [1, 2]. The first *GRF* gene was found in rice (*Oryza sativa*) and was named *OsGRF1*; it was induced by GA3 (gibberellin) [3] and played an essential role in regulating stem elongation. After that, *GRFs* have been reported in various plants, such as *Arabidopsis thaliana*, *Zea mays*, *Medicago truncatula*, and *Brassica rapa* [4–7]. *GRFs* are a large gene family with highly conserved proteins. Most *GRFs* contain unique QLQ (Glu-Leu-Glu, glutamine, leucine, glutamine) and WRC (Trp-Arg-Cys, tryptophan, arginine, cysteine) domains in the N-terminal region [1, 3, 6, 8]. The WRC domain can be combined with the cis-acting regions of downstream genes to regulate their expression. The QLQ domain can interact with the SNH domain in the *GIF* protein to form a transcription activator [3, 6, 8, 9].

Initially, *GRF* was only shown to play a regulatory role in stem and leaf development [10, 11]. Subsequent studies found that *GRF* can also regulate the growth and development of other plant tissues, including flower organ development [12], root development [13, 14], leaf lifespan [15], and plant stress response [16, 17]. Studies have also shown that most members of the *GRF* family have higher expression levels in the meristem. *GRF* genes are usually expressed at higher levels in young tissues but at lower levels in mature tissues [6]. In recent years, studies have shown that *GRF* transcription factors play essential roles in plant growth development and defence responses to biological and abiotic stresses. Further functional classification of the hypothetical downstream targets of *AtGRF1* and *AtGRF3* shows that most of them are involved in defence responses and disease resistance processes [6, 10, 11, 17].

*GIF* proteins are SSXT superfamily genes, a class of plant transcriptional coactivators that are functionally homologous to human SYT transcriptional coactivators [18]. *AtGIF1*, the first member of the *GIF* family, has been used as bait in yeast two-hybrid assays [14]. *GIF* protein has strong transcriptional activity and strong cell division ability [11]. *AtGIF1* is also involved in the control of leaf growth and morphology [14, 19]. *AtGIF2* and *AtGIF3* have similar biological functions to *AtGIF1*, and they play an important role in regulating the cell division ability of plants [6].

*Panax ginseng* is a perennial herbaceous plant with a long growth period. Ginseng must cope with complex environmental changes during its growth and development time, such as extreme temperature and drought, as well as biotic stresses such as pests and diseases [20]. As essential transcription factors in plant growth and development, response to exogenous hormones, and stress resistance, *GRF* and *GIF* transcription factor families have been found in many plant species. For example, in Chinese cabbage (*Brassica rapa L.* spp*. pekinensis*), most of the *BrGRF* genes were induced by GA3 treatment. Moreover, overexpression of *BrGRF8* in Arabidopsis (*A. thaliana*) could increase the sizes of leaves and other organs by regulating cell proliferation [7]. Overexpression of *ZmGRF11*-*ZmGIF2* and *ZmGRF2*-*ZmGIF3* accelerated inflorescence stem growth compared with the wild type [5]. In *Prunus persica*, *PpGRFs* responded to UVB and GA3 treatment and participated in the growth process of new shoot elongation [21]. In *Fragaria vesca*, *FvGRFs* play a potential role in the growth and development of vegetative organs [22]. In callus cells of *Beta vulgaris*, ectopic expression of Arabidopsis *GRF5* accelerated shoot formation and improved transformation efficiency [23]. The molecular characterization of ginseng *GRF* and *GIF* genes has not been studied. Therefore, identifying and analysing the ginseng *GRF* gene is of great significance.

*GRF* and *GIF* may also be involved in controlling the growth and development of ginseng tissues or organs as a class of important transcription factors. This study identified 20 *GRF* and ten *GIF* genes in ginseng. The structural characteristics, phylogenetic relationships, gene duplication events, collinearity, and expression patterns of the *PgGRF* and *PgGIF* genes were also analysed at the genome level, laying the foundation for further study of *PgGRF* and *PgGIF*.

## Result

### Identification and phylogenetic analysis of the *PgGRF* and *PgGIF* gene families

Based on the hidden Markov model (HMM) of the WRC (PF08879) and QLQ (PF08880) domains, a total of 20 *GRF* genes were identified from the ginseng genome, ranging from 346 aa (*PgGRF14* and *PgGRF16*) to 1240 aa (*PgGRF17*) amino acids in length, and the coding sequences (CDSs) of *PgGRFs* ranged from 1041 bp (*PgGRF14* and *PgGRF16*)—3723 bp (*PgGRF17*) in length. In addition, the molecular weights ranged from 38.44 kDa (*PgGRF14*) to 139.81 kDa (*PgGRF17*), and the isoelectric points were between 5.96 (*PgGRF10*) and 9.31 (*PgGRF9*). The pI values of 16 *PgGRF* members were greater than 7, while only *PgGRF3*, *PgGRF10*, *PgGRF17* and *PgGRF20* had pI values less than 7. This may be related to the different effects of *PgGRF* on the growth and development of ginseng (Table S1).

We obtained ten *PgGIF* genes by validating *GIF*’s conserved domain SSXT (PF05030). The amino acid lengths of the *GIF* proteins in ginseng ranged from 176 aa (*PgGIF7*) to 214 aa (*PgGIF5*), and the coding sequences (CDSs) of *PgGRFs* ranged from 531 bp (*PgGIF7*) to 645 bp (*PgGIF5*) in length. In addition, the molecular weights ranged from 18.49 kDa (*PgGIF7*) to 22.97 kDa (*PgGIF5*), and the isoelectric points varied from 5.73 (*PgGIF8*) to 7.94 (*PgGIF1*), thereby indicating that these *GIF* proteins are rich in acidic amino acids (Table S2).

**Fig. 1:**
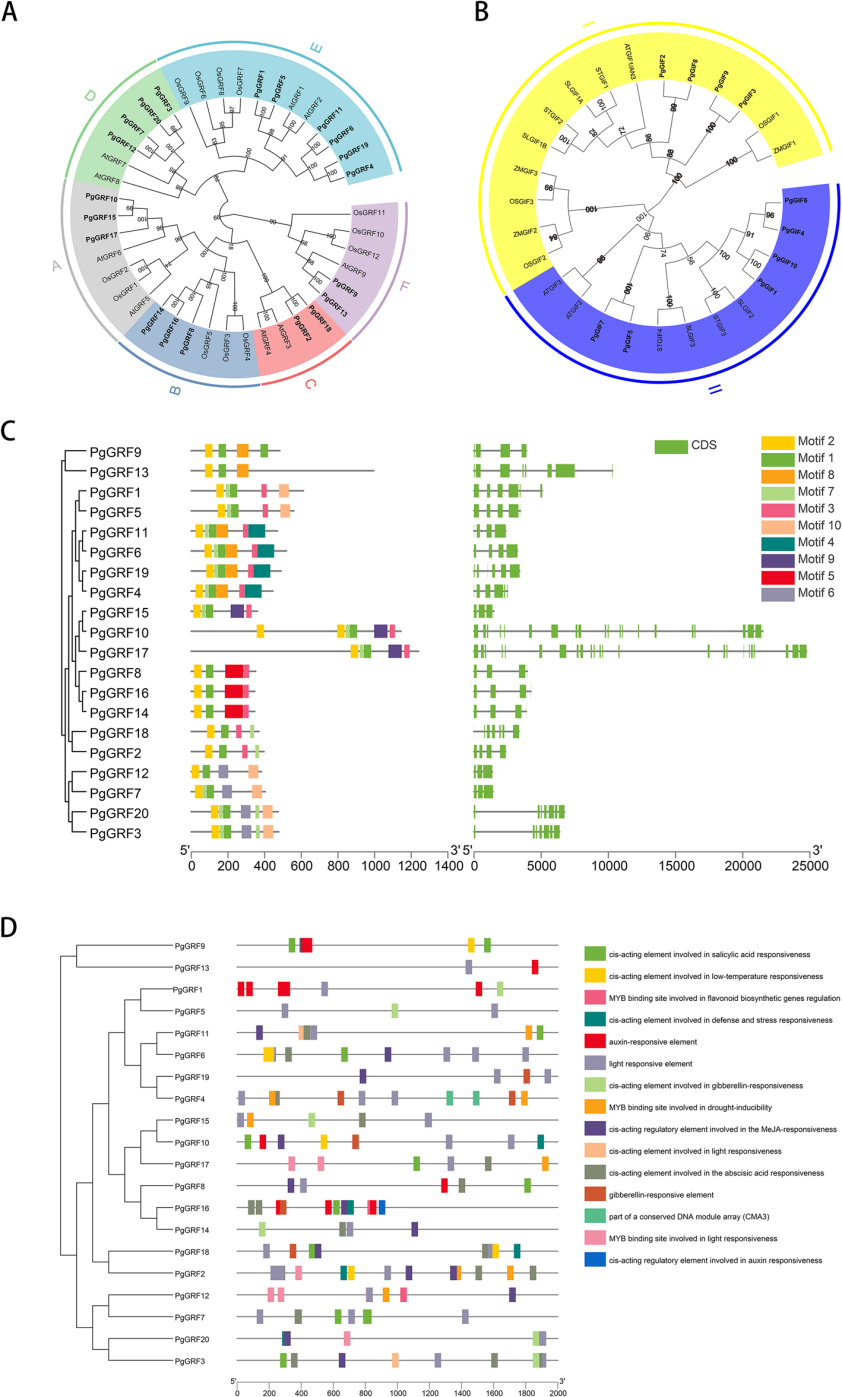
Phylogenetic analysis of PgGRF and PgGIF. **A** Phylogenetic tree based on the growth regulating factors (GRFs) protein family in three plants. At, Arabidopsis; Os, rice; Pg, ginseng. The number stands for the confidence of the branch. **B** Phylogenetic analysis of ginseng GIF proteins and AT, Arabidopsis, OS, rice, ZM, maize, ST, potatoes and SL, tomatoes. **C** Motif compositions of PgGRFs. Conserved motifs in PgGRFs were detected with MEME. Ten different motifs are represented by variously colored boxes. **D** Promoter cis-elements analysis of PgGRFs. The different types of cis-elements are represented by different shapes colors

IQ-TREE was used to construct a maximum likelihood phylogenetic tree (Fig. 1A and Table S3) of *P. ginseng* (20), *O. sativa* (12), and *A. thaliana* (9). All 41 *GRFs* from different species are divided into six clades (A-F). Among the six clades, Clade C is relatively small and contains only four members. In addition, the other three clades (B, D and F) have six members each. In contrast, Clade E contains the most significant number of *GRFs* (twelve), followed by Clade A (seven). Clade B contains only the *GRFs* of rice and ginseng, while Clades C and D, contain only the *GRFs* of Arabidopsis and ginseng. The phylogenetic tree indicated that *PgGRFs* are more closely related to *AtGRFs* than *OsGRFs*, which may be partly because ginseng and Arabidopsis are both dicotyledonous plants [24].

A phylogenetic tree of the *GIF* genes from six species (*A. thaliana*, *O. sativa*, *Z. mays*, *Solanum tuberosum* and *Solanum lycopersicum*) was constructed (Fig. 1B and Table S3). *PgGIFs* and their counterparts were used for the phylogenetic analysis. The ten *PgGIF* proteins were clustered into two clades (I and II): four ginseng *GIFs* (*PgGIF2*, *PgGIF3*, *PgGIF8* and *PgGIF9*) are in Clade I, while Clade II contains *PgGIF1*, *PgGIF4*, *PgGIF5*, *PgGIF6*, *PgGIF7* and *PgGIF10*.

### Gene structure and conserved domain analysis of *PgGRFs* and *PgGIFs*

All *PgGRFs* contained motif 1 and motif 2, annotated as the *GRF*-specific domains WRC and QLQ, respectively (Fig. 1C and Figure S1). Ginseng *GRFs* are divided into six clades, and each *PgGRF* contains three to six conserved motifs. The *PgGRFs* belonging to the same clade have a similar motif composition. Additionally, some motifs only appear in specific clades. For example, motif 9 is unique to Clade A, motif 5 is unique to Clade B, motif 6 is unique to Clade D, and motif 4 and 8 are specific to Clade E. Overall, the gene structure and motif features support the phylogenetic relationship of *PgGRFs*. Similarly, we identified conserved motifs in the ginseng *GIF* gene, as shown in Figure S2 and Figure S3.

The *PgGRF* gene structure shows that the ginseng *GRF* family member genes contain two to 23 introns, most of which contain two to six introns. Most *PgGRFs* have three to seven exons. However, two genes (*PgGRF10* and *PgGRF17*) in Clade A have 21 to 24 exons. Members of the *PgGIF* gene family have three to five exons. The numbers of exons and introns within the same subfamily have high degrees of similarity.

### Promoter cis-element analysis of *PgGRF* genes

The abundant hormone response elements show that *PgGRF* plays an important role in ginseng hormone signal transduction (Fig. 1D and Table S4). These cis-elements, which included ABRE elements (related to abscisic acid); P-box, GARE-motif, and TATC-box (gibberellin response elements); TGA element (auxin response element); CGTCA motif (involved in MeJA reactions) and TGACG motif; TCA element (participates in the salicylic acid reaction), and AuxRR core (participates in the abscisic acid reaction). In addition, defence and stress response elements (TC-rich repeats), light response elements (GT1-motif, Sp1, MRE, ACE), drought-inducing elements (MBS), and low-temperature response elements (LTR) were identified.

Overall, 16 *PgGRFs* (80.0%) had more than one GT1 motif, suggesting that they may respond to light. Thirteen *PgGRFs* (65.0%) had more than one ABRE motif, which suggested that they may respond to abscisic acid. Additionally, 12 *PgGRFs* (60.0%) possessed at least one CGTCA and TGACG motif, which showed the potential of *PgGRFs* to respond to MeJA. In addition, cis-acting elements related to gibberellin were found in 11 *PgGRFs*. We also found five LTRs and six MBSs in *PgGRF* promoter regions, indicating that these genes might play a role in cold and drought.

### Duplication, synteny and evolution analyses of *PgGRF* and *PgGIF* gene members

**Fig. 2:**
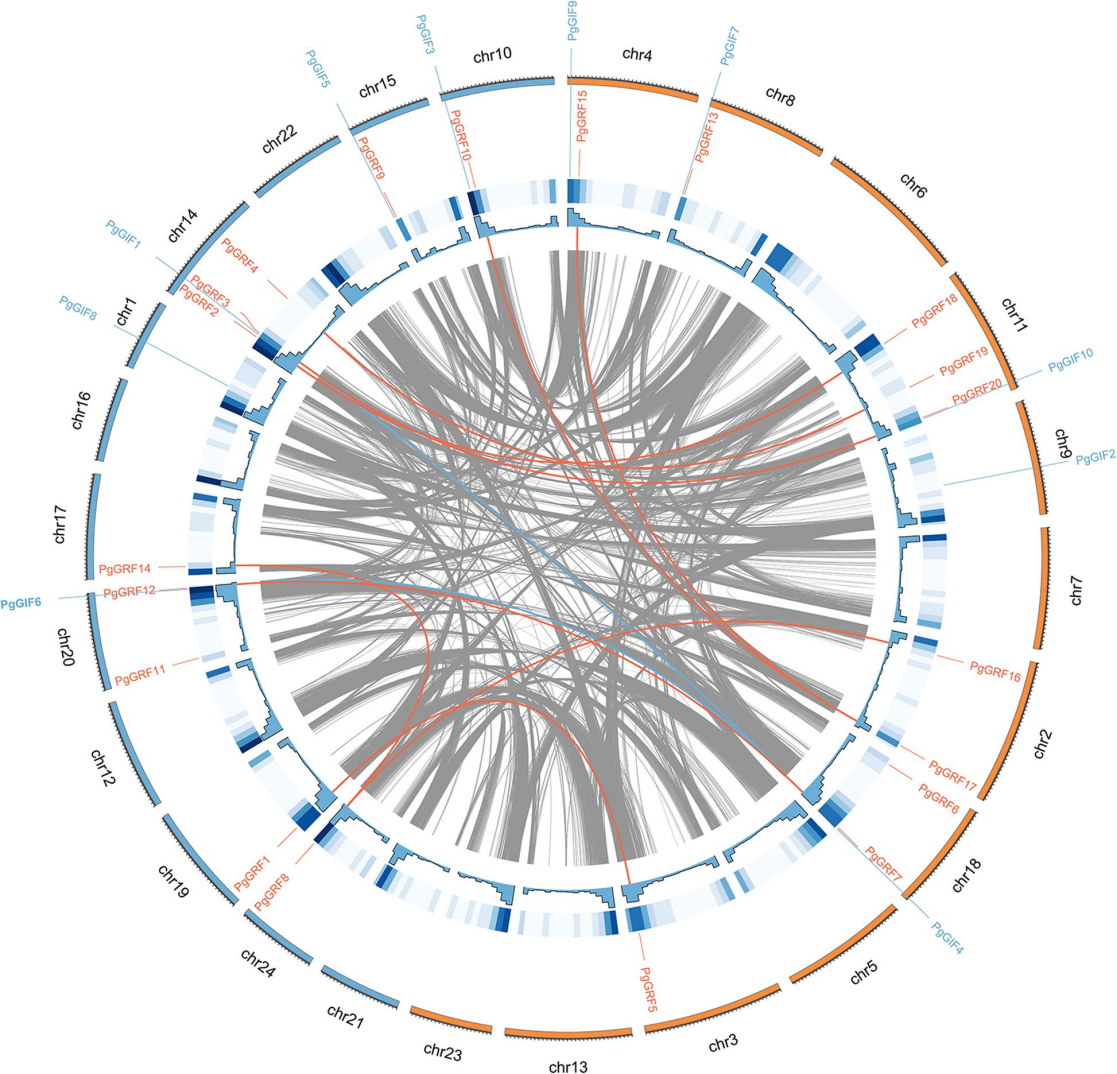
Chromosomal locations and collinearity analysis of the PgGRF and PgGIF gene family. The red lines indicate probably duplicated PgGRF gene pairs. The blue lines indicate probably duplicated PgGIF gene pairs

We visualized and analysed the distribution of *PgGRF* gene family members in the chromosome (Fig. 2). The 20 *PgGRF* family genes were distributed on 13 chromosomes of ginseng. Among them, chr2, 3, 4, 8, 11 and 18 belong to subgenome A, and chr10, 14, 15, 17, 19, 20 and 24 belong to subgenome B. Chr11 and 14 contain three *PgGRF* genes, and chr18 and chr20 have two *PgGRF* genes. Other chromosomes contain one *PgGRF* gene. The ten ginseng *PgGIF* genes were distributed on ten chromosomes. Most genes were mainly distributed at the both ends of chromosomes. It can be seen from the above results that during the genetic evolution of ginseng, *PgGRF* genes were distributed on different chromosomes of ginseng, and the number of genes distributed on each chromosome was different. Gene replication usually mutates genes to derive new functions or divide the functions of ancestral genes crucial to plant adaptation. The expansion of known gene families and the development of new functions contribute to gene replication (tandem and segmental) and differentiation. An intraspecific collinearity analysis showed that eight pairs of *PgGRFs* originated from segmental replication (whole-genome duplication, WGD), accounting for 90% of all ginseng *GRF* family members. In the *PgGIF* family, two pairs of *PgGIFs* originated from segmental replication. Based on the above results, we can infer that WGD events lead to the derivation of new *PgGRF* gene members.

**Fig. 3:**
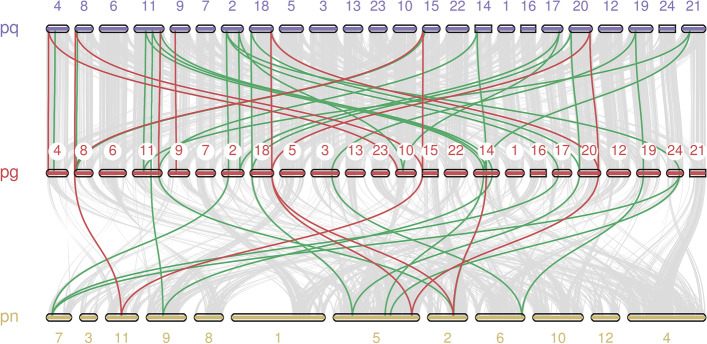
Collinearity relationship of GRF and GIF genes among *P. ginseng*, *P. quinquefolium* and *P. notoginseng*. Identified collinear PgGRF genes are linked by green lines, and PgGIF genes are linked by red lines

To study the collinear relationship between ginseng genes and members of the same family and genus, we analysed the genomic collinearity of *PgGRF* and *PgGIF* in ginseng, *Panax quinquefolium* and *Panax notoginseng* (Fig. 3). The results showed that 19 *PgGRF* genes were collinear with the *P. quinquefolium GRF* gene, and 14 *PgGRF* genes were collinear with the *P. notoginseng GRF* gene. This shows that the *PgGRF* gene family is more closely related to *P. quinquefolium* than *P. notoginseng*. Similarly, the results of interspecific collinearity of ginseng *PgGIF* are shown in Fig. 3. The relationship between *PgGIF* and *P. quinquefolium* was closer.

Repeated genes showed that the Ka/Ks ratios of the *PgGRF* gene family were between 0.276151 and 1.3834. Among them, the Ka/Ks ratios of the *PgGRF1*-*PgGRF5* gene pair were > 1. High Ka/Ks ratios may have been retained to adapt to the environment, indicating that this family may have a complex evolutionary history. The Ka/Ks values of all *PgGIF* gene pairs were < 1. This shows that the *PgGRF* and *PgGIF* gene families may evolve under the action of negative selection (Table S5).

### Expression profiles of *PgGRF* and *PgGIF* genes in different tissues

**Fig. 4:**
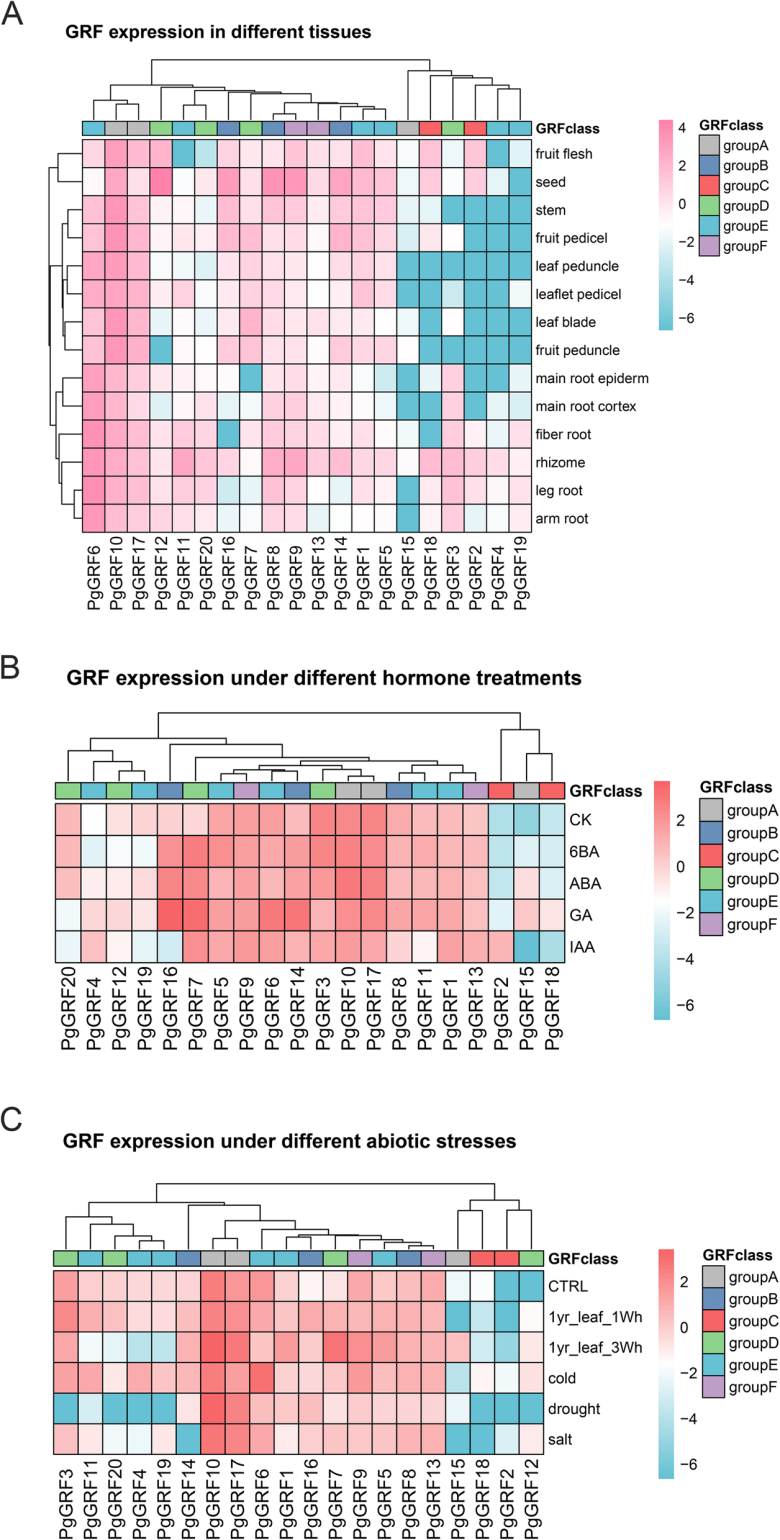
Meta-expression analysis and genome-wide identification of PgGRF and validation of meta-expression patterns. **A** Expression patterns of ginseng GRF Expression in different tissues. **B** Expression patterns of ginseng GRF genes under different hormone treatments. **C** Expression patterns of ginseng GRF genes under different abiotic environmental stresses. Pink and red indicates high expression, and blue indicates low expression

The expression trends of 20 *PgGRFs* in different tissues are shown in Fig. 4A and Table S6. The 14 tissues included fibre root, leg root, main root epiderm, main root cortex, rhizome, arm root, stem, leaf peduncle, leaflet pedicel, leaf lade, fruit peduncle, fruit pedicel, fruit flesh and seed, and expression profiling based on existing transcriptome data. The results showed that only one *PgGRF* gene (*PgGRF15*, FPKM < 1) was not expressed in any tissue. Three *PgGRF* genes, *PgGRF8*, *PgGRF10* and *PgGRF17*, were expressed in 14 tissues (FPKM > 1). The expression patterns of *PgGRFs* are low-level, tissue-distinct and constitutive [25, 26]. Eleven *PgGRF* genes showed low-level expression patterns in all tissues. Only one *PgGRF* gene (*PgGRF11*) was expressed in the rhizome, and three *PgGRFs* (*PgGRF12*, *PgGRF14* and *PgGRF16*) were expressed in seeds. The two *GRF* genes (*PgGRF8* and *PgGRF9*) were only expressed in rhizomes and seeds. In addition, *PgGRF6* was expressed in the root of ginseng, while *PgGRF10* was expressed in the aboveground parts of ginseng (FPKM > 5). The different expression trends of *PgGRFs* indicated that these genes might be involved in various biological processes in different ginseng tissues.

**Fig. 5:**
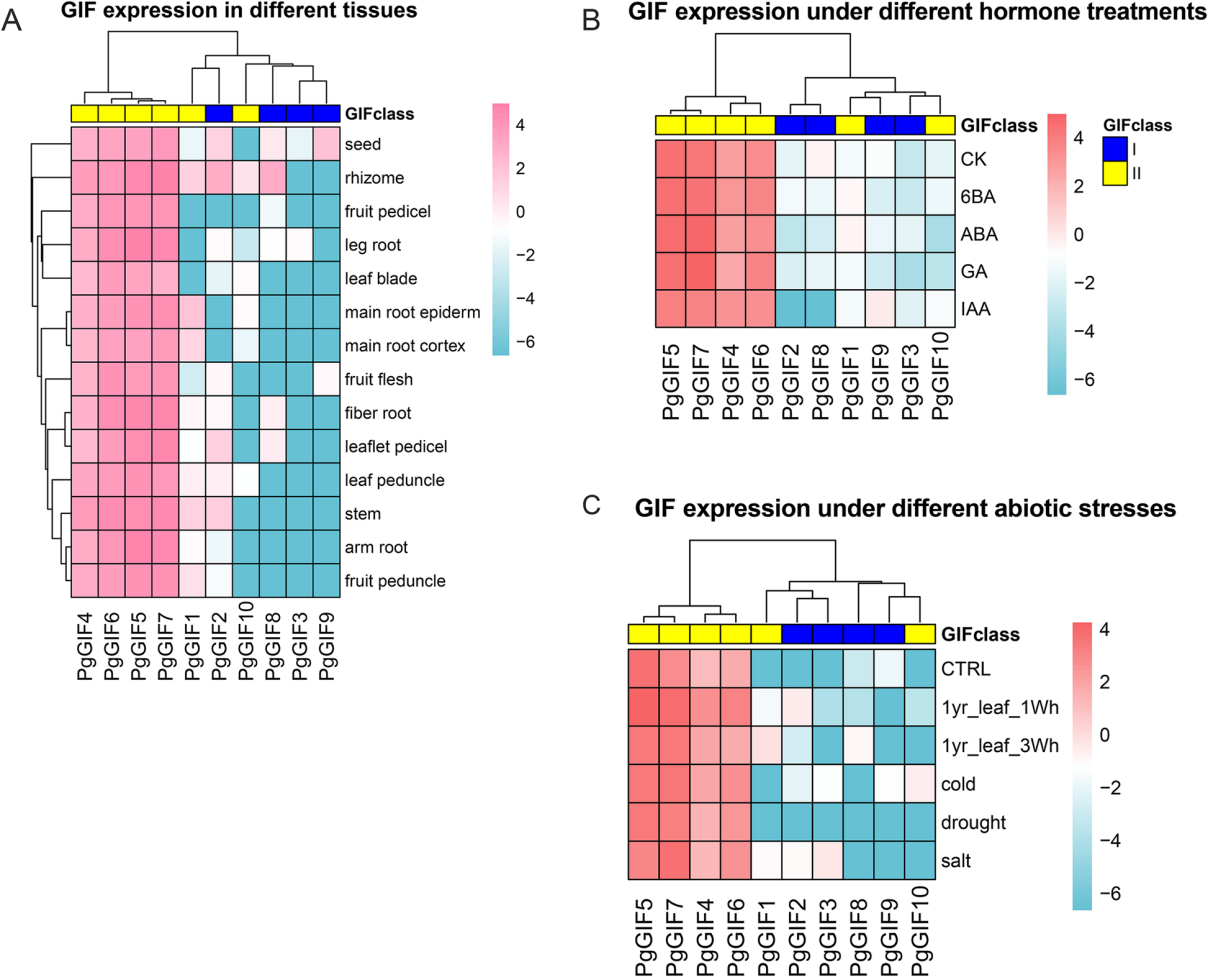
Meta-expression analysis and genome-wide identification of PgGIF and validation of meta-expression patterns. **A** Expression patterns of ginseng GIF in different tissues. **B** Expression patterns of ginseng GIF genes under different hormone treatments. **C** Expression patterns of ginseng GIF genes under different abiotic environmental stresses. Pink and red indicates high expression, and blue indicates low expression

Because the *GIF* protein is involved in the process of plant growth, we preliminarily understood the relationship between *PgGIF* and ginseng growth and development by analysing the expression profiles of the *PgGIF* gene in different tissues (Fig. 5A and Table S6). Among *PgGIF* genes, *PgGIF4*, *PgGIF5*, *PgGIF6* and *PgGIF7* were very prominent. These four genes were highly expressed in 14 ginseng tissues, and the FPKM values were greater than five. Only the *PgGIF3* gene was not expressed in 14 tissues (FPKM < 1). In addition, eight *PgGIF* genes were expressed in rhizomes (FPKM > 1), and seven *PgGIFs* were expressed in seeds (FPKM > 1), which was similar to the *PgGRF* gene expression previously described.

### Transcriptional responses of *PgGRFs* and *PgGIFs* to exogenous hormone treatments

To elucidate the hormone responses of *PgGRF* and *PgGIF* genes, we exposed five-week-old ginseng seedlings to exogenous GA3, 6-BA, IAA, and ABA. The comprehensive expression profiles of genes under hormone treatment are shown in Fig. 4B and Table S7. A small number of PgRGFs showed significantly altered transcriptional levels after hormone treatment. Ten *PgGRFs* in the GA3 treatment, five *PgGRFs* in the IAA treatment, seven *PgGRFs* in the 6-BA treatment, and seven *PgGRFs* in the ABA treatment were upregulated by 1.5-fold or more. The highest fold (fold > 10) inductions in the transcriptional responses to hormones were exhibited by *PgGRF16* (12.4-fold to GA3 and p < 0.05), *PgGRF7* (11.8-fold to GA3 and p < 0.05), *PgGRF2* (39.6-fold to IAA), and *PgGRF15* (58.4-fold to GA3, 35.7-fold to ABA and 7.7-fold to 6-BA). Notably, *PgGRF6* and *PgGRF18* accumulated higher transcription levels in response to GA3 treatment but responded only slightly to other hormones. Across all hormone treatments, *PgGRF*7 was elevated more than fivefold. These results suggest that *PgGRF* genes may function in a manner responsive to hormonal signalling. According to Fig. 5B and Table S7, we found that the expression levels of all *PgGIF* genes did not change significantly under hormone treatment.

### Expression analysis of *PgGRF* and *PgGIF* genes under different abiotic stresses

The published data on different abiotic treatments for ginseng can provide more information for further study of the *PgGRF* and *PgGIF* genes in response to abiotic stress (Figs. 4C and 5C and Table S9). To further explore the response of ginseng *GRF* and *GIF* to low temperature, salt, drought and heat treatments, we analysed the public transcriptome data of ginseng under abiotic stress. Compared with the control group, the expression levels of three ginseng *GRF* genes (*PgGRF4*, *PgGRF6* and *PgGRF11*) increased under cold stress, the expression of one *GRF* gene (*PgGRF16*) increased under drought stress, and the expression levels of two *GRF* genes (*PgGRF7* and *PgGRF16*) increased under salt stress (fold > 2). Similarly, compared with CK, there was no significant change in ginseng *GRF* gene expression after one week of heat treatment, and the expression levels of two *GRF* genes (*PgGRF7* and *PgGRF16*) increased (fold > 2). However, after three weeks of heat treatment, the expression levels of six ginseng *GRF* genes were significantly increased (fold > 2), of which *PgGRF7* (12.7-fold) and *PgGRF15* (5.8-fold) were significantly changed. Similar to the results described in the previous section, ginseng *GIF* genes did not change significantly under cold, drought and salt stress (fold < 2). Compared with CK, the expression levels of *PgGIF4*, *PgGIF6* and *PgGIF7* were increased after one week of heat treatment, and only *PgGIF8* was increased after three weeks of heat treatment (fold > 2), which was different from the change trend of the *PgGRF* gene. The functions of these genes in ginseng need further study.

### Coexpression analysis between *PgGIF* and *PgGRF*

**Fig. 6:**
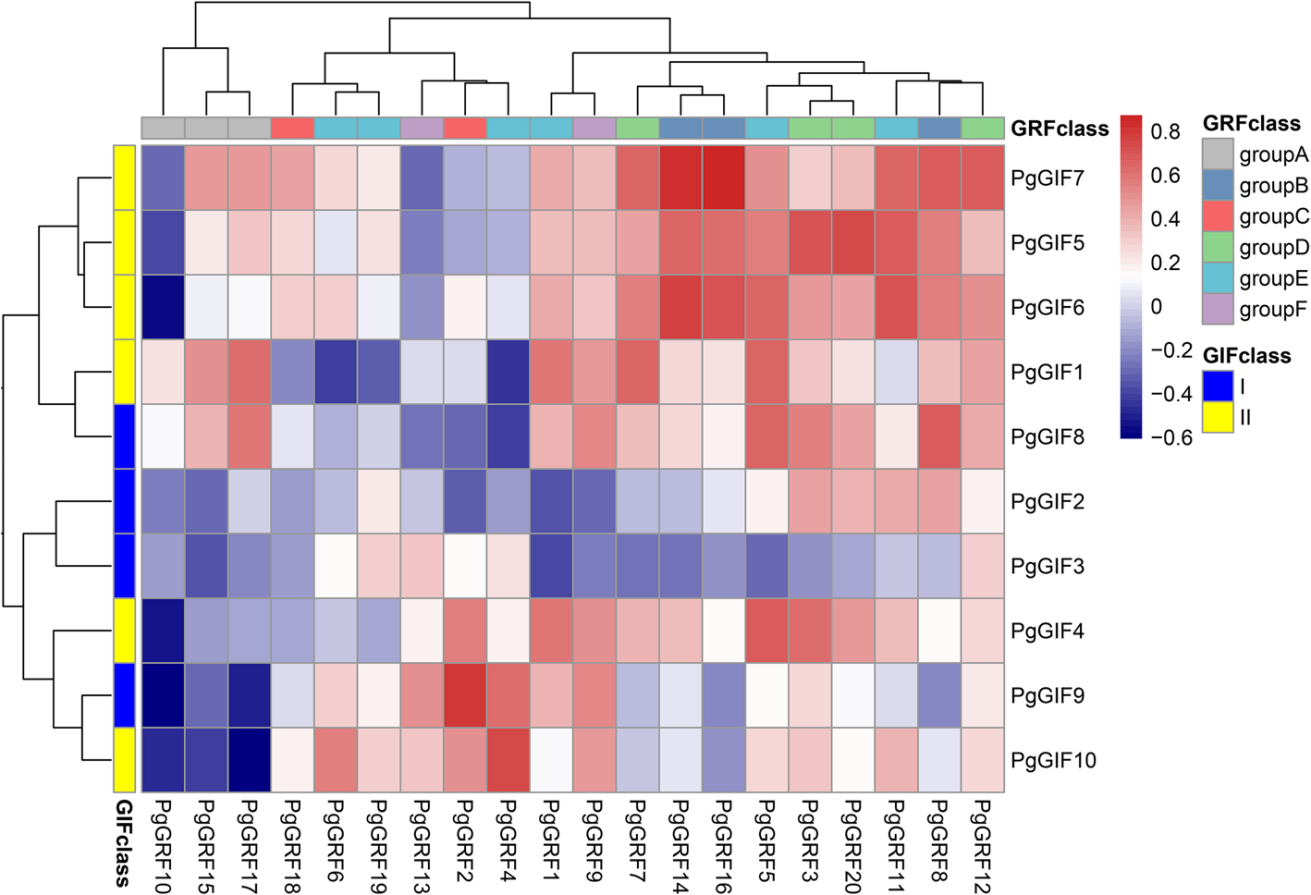
Coexpression between GIF and GRF genes under different hormone treatments and different abiotic environmental stresses. Red indicates high correlation, and blue indicates low correlation

The *GIF1* protein acts as a transcription coactivator to interact with *GRF* proteins in Arabidopsis and rice [27–30]. To further understand whether there is a regulatory relationship between ginseng *GIF* and *GRF* genes, the correlation between *PgGIFs* and *PgGRFs* was analysed (Fig. 6 and Table S10). A total of 27 pairs of *PgGIFs* and *PgGRFs* had correlation r-values greater than 0.6, and we considered them to be strongly correlated. According to the phylogenetic tree analysis results, we focused on five *PgGRFs* (*PgGRF1*, *PgGRF4*, *PgGRF5*, *PgGRF11* and *PgGRF17*) because of their high homology with *AtGRF1* and *AtGRF5*. The expression levels of *PgGIF* and *PgGRF* genes were highly correlated, suggesting a regulatory relationship between these genes.

## Discussion

### The evolution and characterization of *PgGRFs* and *PgGIFs* in ginseng

Studies have suggested that the *GRF* gene family expanded significantly during evolution from lower plants to higher plants, and the number of *GRF* transcription factor genes in land plants ranged from eight to 20 [6]. Only two *GRF* genes have been found in mosses. Genes with regulatory functions are preferentially retained after mass duplications [31]. In previous studies, nine *GRF* genes were found in Arabidopsis, with a genome size of 0.12 Gb; 12 in *O. sativa*, with a genome size of 0.46 Gb; and 14 in *Z. may*, with a genome size of 2.3 Gb. In some recent studies, 30 *GRF* genes were found in wheat (*Triticum aestivum* L*.)*, with a genome size 17 Gb, 8 in *H. vulgare*, with a genome size of 4.5 Gb; and 20 in *Populus. trichocarpa*, with a genome size of 0.48 Gb. We identified 20 *GRF* transcription factors in the ginseng genome in the present study, with a genome size of 2.98 Gb [20]. This result indicated that the number of *GRF* genes was not associated with genome size.

*GIF* acts as a transcriptional coactivator and can form a complex with *GRF*. In our study, we identified ten *GIF* genes in ginseng. *GIF* exists in most eukaryotic organisms, such as embryophytes and metazoans, but is not present in fungi and protists, suggesting that *GIF* genes possess ancient origins [32]. For embryophytes, *M. polymorpha* has only one *GIF* gene, and both *P. patens* and *S. moellendorffii* have four. The basal angiosperm *Amborella trichopoda* has two *GIF* genes. Monocots such as rice and maize have three *GIF* genes, and wheat has four. Dicotyledons, such as tomato (*S. lycopersicum*), have four, and Chinese cabbage has five. Although research on the *GIF* gene family is deepening, no rule has been identified regarding the numbers of *GIF* genes in eudicots or monocots. Similarly, the number of *GIF* genes between annuals and perennials has no obvious rule. For example, the annual plant *G. max* has 11, while *Z. mays* has three. Among the biennial plants, *B. rapa* has five and *Beta vulgaris* has three, while among the perennial plants, *P. trichocarpa* has six, and *Theobroma cacao* has three [27].

Previous studies have reported that the expansion of the *GRF* and *GIF* families mainly occurs through gene duplication, especially large-scale duplication (i.e., whole-genome duplication or fragment duplication), to enhance plant adaptation to environmental changes [27, 33, 34]. This phenomenon exists in soybeans and many other plants. For example, soybean has 22 *GRF* genes. There were also two WGD events (58 million and 13 million years ago) during the evolution of the soybean (*Glycine max*) genome [24]. However, there are only ten *GRF* genes in *Medicago*, and the genome experienced only one WGD event 58 million years ago [35]. Ginseng underwent two whole-genome duplication (WGD) events between 2.2 million and 28 million years ago [20, 36]. In fact, TFs are usually preserved after WGD events [37]. In our study, both ginseng *GRF* and *GIF* were mainly families expanded by WGD events [24, 27]. Therefore, ginseng has more *GRF* and *GIF* genes, which are closely related to the WGD event of ginseng.

According to the phylogenetic results (Fig. 1), 20 *GRF* members in ginseng were clustered into six clades; this evolutionary relationship is similar to previous *GRF* taxonomic studies [24], and the homology of ginseng *GRF* and Arabidopsis *GRF* is higher than that of rice *GRF*, which may be related to the fact that ginseng and Arabidopsis are dicotyledonous plants. Gain or loss events of exons or introns provide structural and functional differences [38]. From the gene structure of each group of responses, most of the *PgGRF* genes have similar structures, and most of the *PgGRF* genes have two to four introns/exons, which are similar to those of rice and Arabidopsis [6, 8]. Previous reports showed that suppressed *OsGRF3*, *OsGRF4* and *OsGRF5* in clade B could cause plant dwarfing, delayed growth and inflorescence formation [39]. The *AtGRF7* gene in clade D is involved in osmotic stress [1]. Overexpression of *AtGRF9* can produce smaller leaves and petals [40]. We speculate that the *PgGRF* genes in these three clades also have the same function.

In Arabidopsis, mutants of *AtGIF1* can change leaf shape [11]. *AtGIF1* interacts with *AtGRF1*, *AtGRF2*, *AtGRF4*, *AtGRF5* and *AtGRF9* through its conserved QLQ domain [11, 41]. Three ginseng *GRF* genes (*PgGRF10*, *PgGRF15*, and *PgGRF17*) in branch A are highly homologous to *AtGRF5*, so we believe they may have similar functions, such as regulating cell proliferation, improving plant tolerance or participating in stem growth and delaying leaf senescence [42]. Studies of *AtGRF* proteins have shown that *AtGRF5* only tightly interacts with *AtGIF1* [11, 43]. Therefore, we speculate that *PgGRF10*, *PgGRF15* and *PgGRF17* can also bind to the SNH conserved domain of *PgGIF* proteins to obtain a better gene expression effect.

### *PgGRFs* and *PgGIFs* are involved in the growth and development of ginseng

Previous studies have confirmed that *GRFs* are expressed in different tissues, usually in growth areas where cell proliferation occurs, such as germinating seeds, calli and shoots [1, 6, 11–13, 30]. The expression levels of *PgGRFs* in 14 tissues were calculated based on FPKM. As shown in Fig. 4A, *PgGRFs* had different expression patterns in 14 tissues. These tissue-specific expression patterns suggest that these *PgGRFs* may be involved in tissue-specific developmental and signalling processes. Some *PgGRFs* were highly expressed in other tissues, such as leaf blade, fruit peduncle, stem and fruit pedicel (FPKM > 10). This means that the vast majority of *PgGRFs* are involved in the growth and development of ginseng. The expression levels of *GRF* genes were significantly higher in actively growing tissues than in mature tissues, and *GRF* transcription levels decreased with plant senescence [6, 8, 41]. The expression level of *AtGRF* decreased with increasing plant age [6, 44]. *GRF* genes in rice are strongly expressed in shoots and immature leaves [8]. The median FPKM values of fibre roots, leg roots, fruit pedicels, rhizomes and seeds were higher than those in other tissues, indicating that *PgGRF* may be involved in more physiological processes in these tissues. *PgGIFs* may be transcriptional coactivators of *PgGRFs* because the expression profiles of *PgGIFs* showed a similar trend to *PgGRFs*. *GRF* and *GIF* proteins positively regulate leaf size by promoting cell expansion and proliferation [11, 15, 41, 45]. The specific functions of *PgGRF* and *PgGIF* in different parts of ginseng need further research and discovery.

### *PgGRFs* and *PgGIFs* are involved in abiotic stresses responses of ginseng

Previous studies have shown that plant hormones regulate many physiological processes, such as growth, differentiation, and development. The first *GRF* was *OsGRF1*, found in gibberellin-treated rice [3]. *GRF* acts as an upstream repressor of the KNOX gene that inhibits GA3 biosynthesis, and GA3 treatment leads to the upregulation of *GRF* [46]. GA3 treatment increased the expression of some *PgGRF* genes in tobacco and some *AhGRF* genes in peanuts [33, 47]. However, *GRFs* in Arabidopsis were not significantly affected by GA3 [6, 8]. In our study, we tested the responses of the *PgGRF* genes to various hormones. Eight of the 20 *PgGRFs* were upregulated by twofold or more in GA3 treatment. In addition, the expression levels of most *PgGRF* genes in this study were maintained or enhanced under 6-BA and ABA treatment, while the expression levels of most genes decreased under IAA treatment. *PgGRF* genes showed significant differential expression, suggesting that these *PgGRFs* may play different roles in hormonal feedback regulation. Various hormone-related cis-elements are found in the *PgGRF* promoters. The results showed that six of the ten *PgGRF* genes upregulated in the GA3 treatment group contained P-box, GARE-motif, and TATA-box elements, and six of the seven *PgGRF* genes upregulated in the ABA treatment group contained ABREs. We therefore believe that cis-acting element analysis can predict the responses of certain transcription factors to hormonal treatment. *PgGRF* and *PgGIF* genes may regulate physiological processes through interactions with molecular and hormonal signals. Further qRT-PCR analysis was carried out for genes with significantly increased expression under different hormone treatments. The results showed that the expression patterns of 12 genes (*PgGRF2, PgGRF4, PgGRF6, PgGRF7, PgGRF14, PgGRF16, PgGRF18, PgGIF1, PgGIF3, PgGIF7, PgGIF9, PgGIF10*) were basically consistent with RNA-seq data.

During the long evolutionary process, plants have acquired a series of signalling pathways and defence systems to resist environmental stresses, and transcription factors play crucial roles in plant responses to various environmental stresses. *GRF* transcription factors play important roles in plant growth by coordinating stress responses and defence signals [16, 17, 48]. For example, under stress conditions, overexpression of Arabidopsis *AtGRF7* increases resistance to drought stress [1]. *AtGRF1* and *AtGRF3* coordinate plant growth, defence signals, and stress responses [14, 49]. Transcriptome data showed that 13 *PgGRFs* were upregulated under cold treatment, five *PgGRFs* were upregulated under drought stress, six *PgGRFs* were upregulated under salt stress, and ten *PgGRFs* were upregulated under a one-week heat treatment. The expression levels of ten *PgGRFs* increased under a three-week heat treatment. Three *PgGIF* genes (*PgGIF4*, *PgGIF6*, and *PgGIF7*) responded to all treatments, indicating that they may play important roles in the response of ginseng to abiotic stress. This study found that the expression levels of the *PgGRF* and *PgGIF* genes had similar trends under cold, drought and salt stresses. Their gene changes were not very significant under these three abiotic stresses, and the genes were relatively more responsive to cold stress. Although there were significant changes in the two family members in the heat treatment group, the *PgGRF* gene was more responsive to a three-week heat treatment, while *PgGIF* gene was more responsive to a one-week heat treatment. These findings suggest that both *GRF* and *GIF* genes may be involved in biological processes related to abiotic stress responses, especially in plant responses to changes in temperature conditions. The responses and functions of *PgGRF* and *PgGIF* in ginseng under environmental stress need further verification.

### Regulatory relationship between *PgGRFs* and *PgGIFs*

Functional studies have shown that *AtGIF1* interacts with six *GRF* proteins in Arabidopsis [11, 15, 41], while *OsGIF1* interacts with three *GRF* proteins [29, 48, 50]. *GIF* genes may also mediate different plant growth and development pathways by interacting with different *GRF* genes [19]. In the above results, we focus on five *PgGRFs* (*PgGRF1*, *PgGRF4*, *PgGRF5*, *PgGRF11* and *PgGRF17*), which have a strong correlation with at least one *PgGIF* (r > 0.6), which also means that they may have a synergistic effect with *PgGIF* in ginseng. Phylogenetic tree analysis showed that *PgGIF8* and *PgGIF9* had homology with *AtGIF1*/AN3 among the eight *PgGIF* genes with a strong correlation with *PgGRF*, and they interacted with different *PgGRF* genes, which indicated the difference in *PgGIF* genes in the evolution process [19]. Coexpression analysis showed that 27 pairs of *PgGIFs* and *PgGRFs* had high correlations (r > 0.6), suggesting that they may be regulated by the same TF [19]. In addition, it has been reported that several *GRF* proteins in rice [51] and maize [52] are located downstream of the *GIF* gene, and increasing the expression of the *GIF* gene can increase the transcription level of the *GRF* gene. The specific functions and modes of action of the different *GRFs* and *GIFs* in ginseng may require further research.

## Conclusion

In our study, the genome-wide identification and analysis of *GRF* and *GIF* TFs in ginseng and their induction in different tissues, upon exposure to different hormones, and in response to different abiotic stresses were performed. The results of coexpression studies indicated potential interactions between *PgGRFs* and *PgGIFs*. Our results lay a foundation for further research on the roles of *PgGRFs* and *PgGIFs* in the growth and development of ginseng, provide valuable information for the functional study of transcription factors in ginseng, and provide a theoretical basis for ginseng variety selection.

## Materials and methods

### Plant cultivation and treatment

Hormone treatment: sown ginseng seeds in a culture bowl cultivate at room temperature at 25 °C, with a relative humidity of about 60%, and 16 h light/8 h dark. When the seedlings (JIMEI Ginseng) grow for five weeks and have three real leaves, spray with different hormones, including ABA: 50 mM, IAA: 10 mM, 6-BA: 75 mM, GA3: 100 mM and the control group is treated with distilled water. The treatment time is five hours, the biological repetition is three times, and the seedlings are collected and stored at -80 °C. The environmental stress analysis uses public data [20].

### GRF and GIF sequence retrieval and identification

The candidate GRF and GIF genes were firstly obtained from the Ginseng Genome Data resource [36]. Hidden Markov Models (HMMs) for GRF and GIF conserved domains WRC (PF08879), QLQ (PF08880) and SSXT (PF05030) were extracted from the Pfam database (http://pfam.xfam.org). The GRF and GIF genes retrieved from the ginseng genome were detected by HMMER 3.2.1 software, and the E-value threshold was 10^−2^. All candidate PgGRFs and PgGIFs were further validated using the SMART data resource (http://smart.embl.de/), NCBI-Conserved Domain Database (CDD) and PlantTFDB (Plant Transcription Factor Database) (http:// planttfdb.cbi.pku.edu.cn) to ensure that they contain both GRF or GIF domains.

### Phylogenetic analysis and gene structure analysis

Mafft (https://mafft.cbrc.jp/alignment/software/) with default parameters was used for multiple alignments of ginseng GRF sequences as well as for multiple alignments of GRFs among other species. The ginseng GRF phylogenetic tree was established by the maximum likelihood method IQ-TREE based on the JTTDCMut + F + R4 model [53], and the nodes were tested 1000 times by bootstrap analysis. Further annotation of the phylogenetic tree results was handled by Evolview (https://evolgenius.info/).

TBtools 1.053 was employed to demonstrate the gene structure [54]. Conserved motifs of PgGRFs were identified using MEME native software (version 4.12.0) in Linux with a maximum of 10 mismatches and an optimal motif width of 6–100 amino acid residues. In addition, theoretical isoelectric point (pI) along with the molecular weight (MW) of PgGRF proteins were predicted by the online Sequence Manipulation Suite (http://www.detaibio.com/sms2/reference.html) [55]. Likewise, the GIF gene was also analyzed using the method described above.

### Cis-acting elements analysis

The sequence of 2000 bp upstream of the start codon of PgGRFs and PgGIFs was obtained for promoter analysis. Use PlantCARE (http://bionformatics.psb.ugent.be/webtools/plantcare/html) to predict cis-acting elements in the promoter region and use PlantTFDB software (http://planttfdb.cbi.pku.edu.cn/) online Predict the distribution of promoter transcription factor binding sites (p-value ≤ 1e^−6^).

### Meta-expression analysis

To analyze gene expression among different tissues and responses to different abiotic treatments. We retrieved RNA-Seq datasets from 14 different tissues from NCBI (accession number PRJNA302556) [56] and 15 RNA-Seq datasets for abiotic treatment (No.24–38 in ginseng transcriptome data resource, http://ginsengdb.snu.ac.kr/transcriptome.php) from Ginseng Genome Data Resource (http://ginsengdb.snu.ac.kr/) were retrieved. The clean reads were aligned to the ginseng genome using Hisat2 software. Hisat2, StringTie and ballgown were used to assemble and calculate the expression value for each transcript.

The hormone treatment cDNA libraries were established in a previous study [57]. These 15 cDNA libraries were finally sequenced on HiSeq 2500 (Illumina) with the PE125 strategy. The FRKM was calculated using the same protocol for the other 16 RNA-Seq datasets. The heatmap was generated by the R package “Heatmap”.

### Quantitative-real time PCR analysis

Total RNA was prepared from samples using a *EasyPure* Plant RNA Kit (TransGen Biotech). RNase-free DNase I (TransGen Biotech) was used in the extraction process to remove DNA contamination. Both the concentration and the quality of the RNA samples were evaluated with a NanoPhotometer N50 (Implen, GER). Use the *PerfectStart* Uni RT&qPCR Kit (TransGen Biotech) to reverse transcribe RNA into cDNA and perform two-step Quantitative Real-time PCR. qRT-PCR was performed using a Stratagene Mx3000P SYBR-GREEN I Master (Agilent, USA). *β*-Actin gene is used as internal control [58]. For the data analyses, the 2^−ΔΔCT^ method was used for calculating the relative expression of PgGRF and PgGIF genes [59]. Primers for qRT-PCR were synthesized by Sangon Biotech (ShangHai, China), and sequences are listed in Supplementary Table S8.

### Chromosomal location, duplication, synteny and evolution analyses

The MCScanX program was used for inter- and intra-species collinearity analysis of proteins with an E value of 1e^−5^, and the Duplicate Gene Classifier script in the MCScanX program was used to quantify various forms of duplication (WGD or segmental, tandem, dispersed and proximal duplication) and visualized by the Circos [60, 61].

Using KaKs-Calculator-2.0 calculates the non-synonymous replacement rate (Ka) and synonymous replacement rate (Ks) of replicated gene pairs and analyzes the environmental selection pressure through the Ka / Ks ratio [62].

### Correlation coefficient analysis between PgGRFs and PgGIFs

The FPKM value of PgGRF and PgGIF under hormone-induced and abiotic environmental stress conditions was used to calculate the Pearson’s correlation between the two gene families by the R package “Hmisc”.

## Supplementary Information

**Additional file 1: Figure S1. **GRF conserved motif structure of *ginseng.*

**Additional file 2: Figure S2.** Gene structure of ginseng GIF family.

**Additional file 3: Figure S3. **GIF conserved motif structure of *ginseng.*

**Additional file 4: Figure S4. **expression analyses of PgGRF and PgGIF genes under different hormone treatments conditions analyzed by qRT-PCR. CK: control sample. ABA: 50 mM, IAA: 10mM, GA3: 100 mM. Data were normalized to *β*-actin gene and vertical bars indicated standard deviation.

**Additional file 5: Table S1.** Identification and characterization of PgGRF genes in *P. ginsneg*. **Table S2.** Identification and characterization of PgGIF genes in *P. ginsneg*. **Table S3.** GRF genes found in Arabidopsis thaliana and *Oryza sativa*. And protein sequences of GIFs from *A. thaliana*, *O. sativa*, *Z. mays*, *S. tuberosum* and *S. lycopersicum*. **Table S4. **The detailed information of cis-elements in the promoter regions of PgGRF genes in *P. ginseng*. **Table S5.** The ka/ks values of PgGRF and PgGIF genes in *P. ginseng*. **Table S6.** The FPKM values of PgGRF and PgGIF genes in different *P. ginseng* tissues. **Table S7.** The FPKM value of PgGRF and PgGIF genes transcriptional response to exogenous hormones treatments in *P. ginxeng*. **Table S8.** Primers for qRT-PCR of candidate genes in *P. ginseng* GRF and GIF gene families. **Table S9. **The FPKM value of PgGRF and PgGIF genes transcriptional response to abiotic stresses in *P. ginseng*. **Table S10.** The FPKM value is the correlation between PgGRF and PgGIF genes in *P. ginseng*.

## Data Availability

The raw RNA-Seq data of 14 *P. ginseng* tissues were downloaded from the NCBI Sequence Read Archive ( https://www.ncbi.nlm.nih.gov/bioproject/PRJNA302556). The raw RNA-Seq datasets of drought, salt and cold treatment were downloaded from Ginseng Genome Database (http://ginsengdb.snu.ac.kr/download.php?filename=DSC.tar.gz), and the heat stress was downloaded from Ginseng Genome Database ( http://ginsengdb.snu.ac.kr/download.php?filename=Heat.tar.gz). All other data generated or analysed in this study are included in this article and its additional files.

## Abbreviations

GRF: Growth-regulating factors
GIF: GRF-interacting factors
TF: Transcription factor
WGD: Whole-genome duplication
MW: Molecular weight
pI: Isoelectric point
aa: amino acid
kDa: Kilodaltons
6-BA: 6-Benzylaminopurine
ABA: Abscisic acid
GA3: Gibberellin
IAA: Indole-3-acetic acid
MeJA: Methyl Jasmonate
SA: Salicylic acid
CDS: Coding sequences
MEME: Multiple expectation maximization for motif elicitation
chr: Chromosome
FPKM: Fragments per kilobase of transcript per million mapped fragments
HMM: Hidden Markov model
MEGA: Molecular evolutionary genetics analysis
MYA: Million years ago
Ka: Non-synonymous
Ks: Synonymous
