# Supplementary figures and images for: Whole-genome identification and expression profiling of growth-regulating factor (GRF) and GRF-interacting factor (GIF) gene families in Panax ginseng
Source: BMC Genomics. 2023 Jun 16;24:334. doi: 10.1186/s12864-023-09435-w (PMC10276473; doi:10.1186/s12864-023-09435-w)

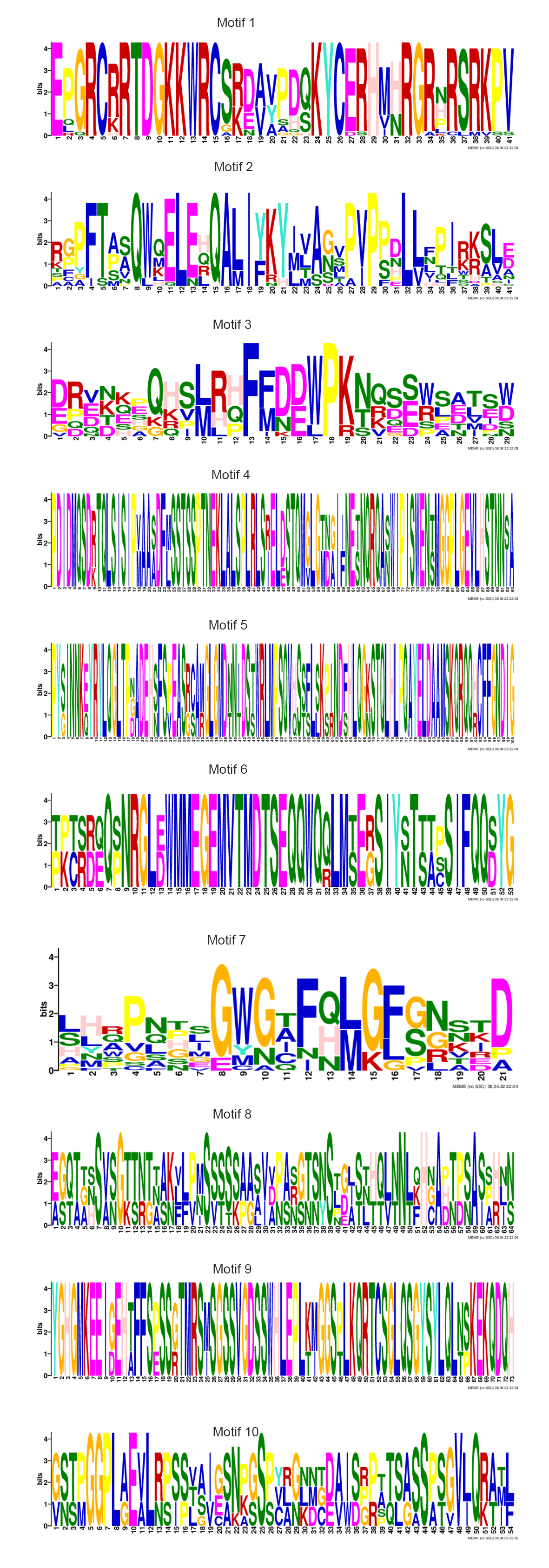

Supplement: Supplementary file 1 — Additional file 1: Figure S1. GRF conserved motif structure of ginseng. [file 12864_2023_9435_MOESM1_ESM.png]

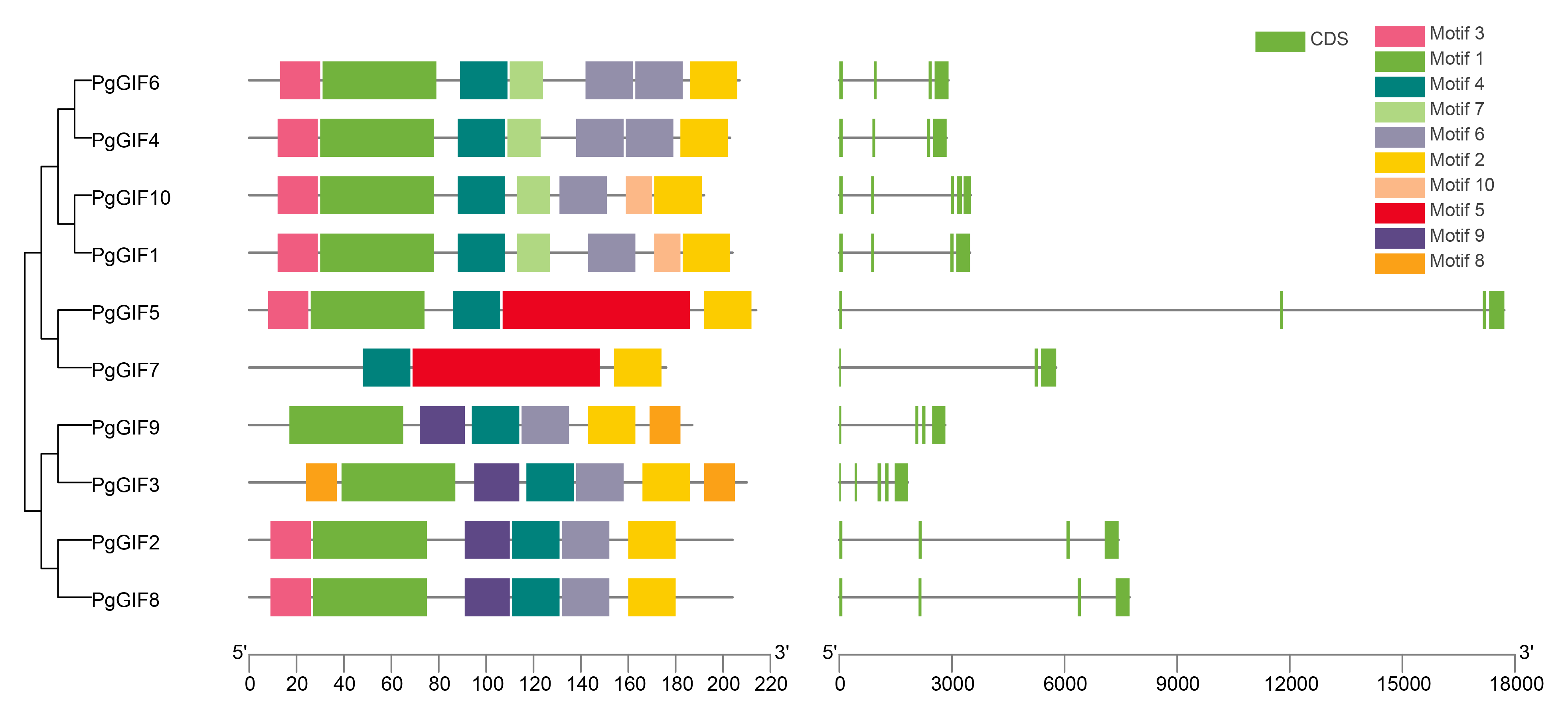

Supplement: Supplementary file 2 — Additional file 2: Figure S2. Gene structure of ginseng GIF family. [file 12864_2023_9435_MOESM2_ESM.png]

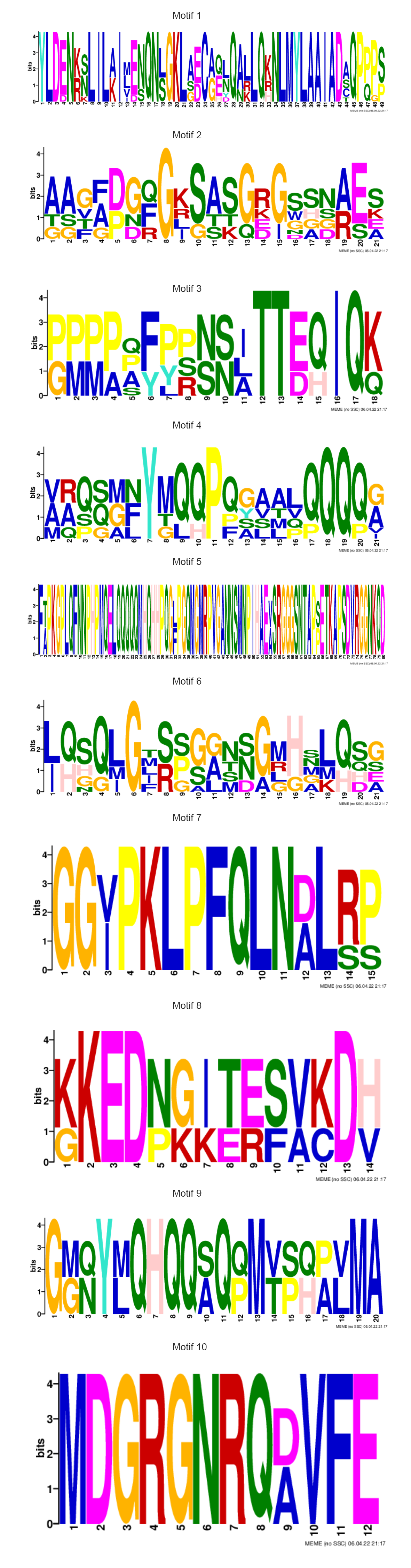

Supplement: Supplementary file 3 — Additional file 3: Figure S3. GIF conserved motif structure of ginseng. [file 12864_2023_9435_MOESM3_ESM.png]

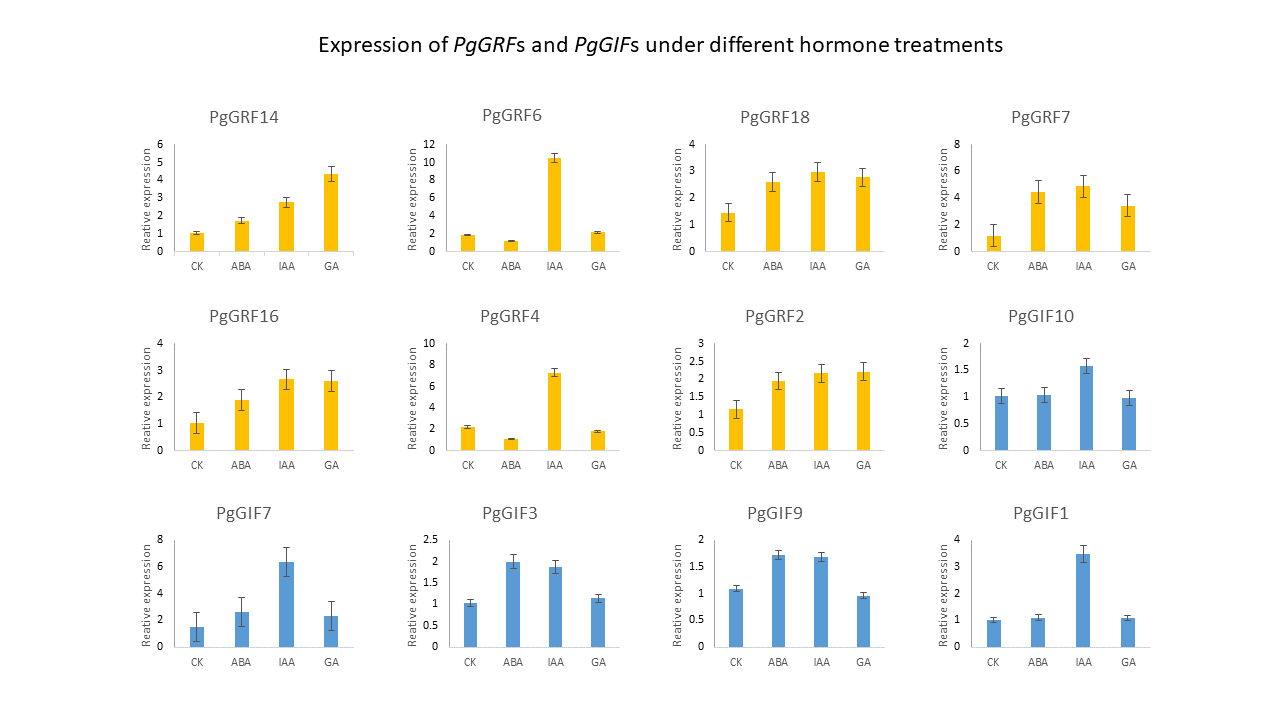

Supplement: Supplementary file 4 — Additional file 4: Figure S4. expression analyses of PgGRF and PgGIF genes under different hormone treatments conditions analyzed by qRT-PCR. CK: control sample. ABA: 50 mM, IAA: 10mM, GA3: 100 mM. Data were normalized to β-actin gene and vertical bars indicated standard deviation. [file 12864_2023_9435_MOESM4_ESM.png]
